# Characterization of the Cardiac Renin Angiotensin System in Oophorectomized and Estrogen-Replete mRen2.Lewis Rats

**DOI:** 10.1371/journal.pone.0076992

**Published:** 2013-10-25

**Authors:** Hao Wang, Jewell A. Jessup, Zhuo Zhao, Jaqueline Da Silva, Marina Lin, Lindsay M. MacNamara, Sarfaraz Ahmad, Mark C. Chappell, Carlos M. Ferrario, Leanne Groban

**Affiliations:** 1 Department of Anesthesiology, Wake Forest School of Medicine, Winston-Salem, North Carolina, United States of America; 2 Department of Hypertension and Vascular Research Center, Wake Forest School of Medicine, Winston-Salem, North Carolina, United States of America; 3 Department of Internal Medicine/Nephrology, Wake Forest School of Medicine, Winston-Salem, North Carolina, United States of America; 4 Department of Surgery, Wake Forest School of Medicine, Winston-Salem, North Carolina, United States of America; University of Padua, Italy

## Abstract

The cardioprotective effects of estrogen are well recognized, but the mechanisms remain poorly understood. Accumulating evidence suggests that the local cardiac renin-angiotensin system (RAS) is involved in the development and progression of cardiac hypertrophy, remodeling, and heart failure. Estrogen attenuates the effects of an activated circulating RAS; however, its role in regulating the cardiac RAS is unclear. Bilateral oophorectomy (OVX; n = 17) or sham-operation (Sham; n = 13) was performed in 4-week-old, female mRen2.Lewis rats. At 11 weeks of age, the rats were randomized and received either 17 β-estradiol (E2, 36 µg/pellet, 60-day release, n = 8) or vehicle (OVX-V, n = 9) for 4 weeks. The rats were sacrificed, and blood and hearts were used to determine protein and/or gene expression of circulating and tissue RAS components. E2 treatment minimized the rise in circulating angiotensin (Ang) II and aldosterone produced by loss of ovarian estrogens. Chronic E2 also attenuated OVX-associated increases in cardiac Ang II, Ang-(1–7) content, chymase gene expression, and mast cell number. Neither OVX nor OVX+E2 altered cardiac expression or activity of renin, angiotensinogen, angiotensin-converting enzyme (ACE), and Ang II type 1 receptor (AT1R). E2 treatment in OVX rats significantly decreased gene expression of MMP-9, ACE2, and Ang-(1–7) mas receptor, in comparison to sham-operated and OVX littermates. E2 treatment appears to inhibit upsurges in cardiac Ang II expression in the OVX-mRen2 rat, possibly by reducing chymase-dependent Ang II formation. Further studies are warranted to determine whether an E2-mediated reduction in cardiac chymase directly contributes to this response in OVX rats.

## Introduction

Left ventricular diastolic dysfunction (LVDD) of aging is heterogeneous, but a higher prevalence of the condition in postmenopausal women postulates a link between estrogen deficiency and LVDD [1], [2]. Results of previous studies indicated that loss of estrogen is associated with development of hypertension and left ventricular hypertrophy [3], [4], [5], two known risk factors for LVDD [6], [7]. Because LVDD might contribute to the progression of heart failure (HF)— by limiting cardiac output reserve, accelerating neuroendocrine activation, increasing symptoms, and by promoting physical inactivity, deconditioning, and frailty [8], [9], [10]—there is a significant need to halt the progression of LVDD after menopause. Current pharmacological approaches have met with minimal success [11], [12]; alternative therapies that achieve the cardiovascular benefits of estrogen replacement therapy without its side effects and contraindications are needed. Experimental evidence shows that estrogen deprivation and replacement affect remodeling of the cardiomyocyte and extracellular matrix, ultimately modifying lusitropic function and ventricular compliance [3], [4], [5]. These effects have potential as new approaches to controlling the progression of LVDD. However, the mechanisms underlying these benefits remain poorly understood.

Using the female mRen2.Lewis rat, an estrogen-sensitive model that emulates the cardiovascular phenotype of the postmenopausal woman, we previously showed that estrogen depletion by oophorectomy (OVX) causes marked worsening of their hypertension, left ventricular remodeling, diastolic dysfunction, and oxidative stress, as well as increased NADPH oxidase or NOX4 expression in the heart [13], [14], [15], [16], [17], [18]. In this rat model, estrogen replacement limits these adverse effects of ovarian hormone loss [14], [15], [16], [17], [18], in part through deactivation of the circulating renin-angiotensin system (RAS) [13]. Moreover, estradiol (E2) replacement modestly reduces systemic angiotensin-converting enzyme (ACE) activity in postmenopausal women [19], [20], attenuates the conversion of Ang I to Ang II and down-regulates AT1 receptor expression in the kidney [13], [21], [22], [23] and Ang II-induced aldosterone production in female animal models of aging [24]. Low doses of estrogen or AT1 receptor blockade can also attenuate low-grade systemic inflammation and oxidative stress associated with menopause and ovariectomy [25]. This progress in knowledge remains relatively ignored as we have little information regarding translation of these experimental findings to female-specific therapy for LVDD and HF with preserved ejection fraction [11], [12]. In fact, retrospective analyses of large trials suggest that the effects of ACE-inhibitors may be less pronounced in women than in men receiving treatment for hypertension and heart failure [26], [27], [28], [29].

Although cardiac Ang II is critical in the paracrine/autocrine regulation of cardiac function and in the pathophysiologic process of hypertensive heart disease [30], [31], [32], little is known about the influence of estrogen on the cardiac RAS components, particularly ACE and ACE2. The RAS consists of two biochemical arms: one generates Ang II via the catalytic action of ACE on Ang I; the second generates Ang-(1–7) via action of the endopeptidase, neprilysin. Importantly, ACE2 directly converts Ang II into Ang-(1–7) [33], [34], [35]. Although the physiologic role of ACE2 or Ang-(1–7) in the postmenopausal heart is not known, evidence suggests that, by metabolizing Ang II and increasing Ang-(1–7), ACE2 might counterbalance the vasopressor and profibrotic effects of the ACE/AngII/AT1receptor pathway [36], [37], [38], [39], [40], [41], [42], [43].

The present study tested the hypothesis that estrogen replacement with 17 β-estradiol in oophorectomized mRen2.Lewis rats prevented development of diastolic dysfunction and LV remodeling, as previously reported [18], via a shift in the circulating and cardiac RAS from the pro-fibrotic Ang II/AT1R/ACE and aldosterone pathway to the anti-fibrotic, Ang-(1–7)/Mas/ACE2 pathway. We also determined the contribution of chymase to the female mRen2.Lewis cardiac phenotype, because this serine protease is part of an alternative pathway for the generation of Ang II from Ang I, and is released from cardiac mast cells under conditions of stress, including ischemia, volume and pressure overload (e.g., hypertension) [44].

## Methods

### Ethics Statement

This study was carried out in strict accordance with the recommendations in the Guide for the Care and Use of Laboratory Animals of the National Institutes of Health (NIH publication No. 85-23, revised 1996). The protocol was approved by the Institutional Animal Care and Use Committee (IACUC) at Wake Forest School of Medicine (Approved protocol #A08-221, approved 1/20/2009).

### Animals

The OVX-mRen2.Lewis rat is a well-established animal model that emulates the cardiovascular phenotype of the postmenopausal woman, specifically systolic hypertension, left ventricular hypertrophy, impaired relaxation, and elevated cardiac filling pressures [15], [16], [17], [45]. Female heterozygous mRen2.Lewis rats were obtained from the Hypertension and Vascular Research Center Congenic Colony at Wake Forest School of Medicine. Rats were weaned at three weeks of age, and allowed to acclimate to controlled temperature (22±2°C) and light (12 h light/dark cycle) with *ad libitum* access to food and water, in a facility approved by the Association for Assessment and Accreditation of Laboratory Animal Care.

### Experimental protocol

**Figure 1:**
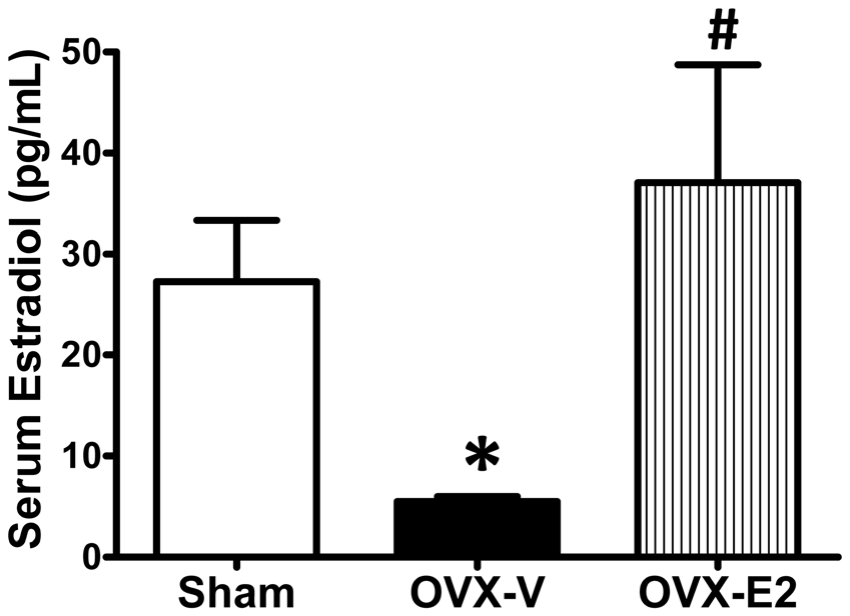
Serum estradiol concentration. Serum estradiol concentration in sham-operated and oophorectomized female mRen2.Lewis rats treated with vehicle or estradiol for 4 weeks. OVX, oophorectomized. Values are means ± SEM; * P<0.05 vs. sham, # P<0.05 vs. OVX-V. n = 7–13/group.

At 4 weeks of age, rats were randomly assigned to undergo either OVX (n = 17) or sham operation (Sham; n = 13) performed under 2% isoflurane anesthesia, as previously described [15], [16], [17]. The adequacy of anesthesia was monitored by observation of slow breathing, loss of muscular tone, and lack of response to surgical manipulation. The success of OVX and subsequent depletion of circulating estrogens were confirmed using a serum estradiol assay (5 pg/mL detection limit; Polymedco, Cortlandt Manor, NY, USA) at the end of the experiment (Figure 1). Once the rats reached 11 weeks of age, the OVX group was randomly divided to receive either E2 (OVX-E2; n = 8) or a placebo control (OVX-V; n = 9). Sixty day-release 17 β-estradiol pellets (36 µg/pellet; Innovative Research of America, Sarasota, FL, USA) or control pellets were implanted subcutaneously in the posterior neck of rats. At 15 weeks of age, rats were euthanized via exsanguination by cardiac puncture, while under ketamine/xylazine anesthesia (ketamine HCL 60 mg/kg and xylazine HCL 5 mg/kg), and all efforts were made to minimize suffering. Whole blood was collected from the abdominal aorta and processed for subsequent determination of estrogen, Ang II, Ang 1–7, and aldosterone. Whole hearts were isolated and dissected to separate the left ventricle (LV), right ventricle, and both atria. Tissue weights were measured with an analytical scale. The LV was cut into pieces for biochemical and histological analyses.

### Analysis of gene expression by quantitative real-time PCR

Real-time PCR was used to detect gene mRNA levels in cardiac tissue. Total RNA was extracted from frozen, pulverized LV tissue from each group using TRIzol Reagent, and processed according to the manufacturer's recommendations. The quality and quantity of RNA samples were determined by spectrometry and agarose gel electrophoresis. Complementary first strand DNA was synthesized from oligo (dT)-primed total RNA, using the Omniscript RT kit (Qiagen Inc, CA). Relative quantification of mRNA levels by real-time PCR was performed using a SYBR Green PCR kit (Qiagen Inc, CA). Amplification and detection were performed with the ABI7500 Sequence Detection System (Applied Biosystems). Only one peak from the dissociation curve was found from each pair of oligonucleotide primers tested. Real-time PCR was carried out in duplicate; a no-template control was included in each run to check for contamination. It was also confirmed that no amplification occurred when samples were not subjected to reverse transcription. Sequence-specific oligonucleotide primers were designed according to published GenBank sequences (www.ncbi.nlm.nih.gov/Genbank) and confirmed with OligoAnalyzer 3.0. The relative target mRNA levels in each sample were normalized to S16 ribosomal RNA. Expression levels are reported relative to the geometric mean of the control group.

### Western blot analysis

LV tissue homogenates were separated by SDS–PAGE and transferred onto membranes, as previously described [15], [16], [17]. Immunoblots were probed using antibodies for AT1R (1∶250; Alomone Labs, Jerusalem, Israel), chymase (1 ug/ml; Bioss, Woburn, MA, USA), MMP-9 (1∶750; Abcam, Inc., Cambridge, MA, USA), ACE2 (1∶1000; Santa Cruz Biotechnology, Santa Cruz, CA, USA), Ang-(1–7) (1∶1000, affinity purified to Ang-(1–7), #5-2010), and mas receptor (1∶5000; Alomone Labs, Ltd, Jerusalem, Israel). Glyceraldehyde-3-phosphate dehydrogenase (GAPDH; 1∶5,000; Cell Signaling, Danvers, MA, USA) was used as a loading control. The bands were digitized using MCID image analysis software (Imaging Research, Inc., Ontario, Canada). Each band was expressed in arbitrary units and normalized to its own GAPDH.

### Immunocytochemical analysis

Immunocytochemical staining of heart sections (4 µm thick) was performed using standard procedures. Formalin-fixed and paraffin-embedded LV sections were deparaffinized, exposed to 3% hydrogen peroxide to block endogenous peroxidase activity, and subjected to antigen retrieval via immersion in citric acid (pH 6.0, 0.01 mol/L) at 95°C for 15 min, followed by slow cooling to 60°C. After treatment with blocking serum, the sections were incubated with antibody overnight at 4°C, rinsed with phosphate-buffered saline, and incubated with biotinylated secondary IgG (Vector Laboratories, Burlingame, CA) for 3 h at 4°C. The primary antibodies included anti-Ang II (1∶10000, IgG Corp, Nashville, TN, USA), chymase (1∶1000, Bioss. Woburn, MA, USA), ACE2 (1∶200, Santa Cruz Biotechnology, Santa Cruz, CA, USA), and Ang-(1–7) (1∶100). Normal serum from the same species, diluted to the same protein concentration as the primary antibody, was used as the negative control. Antibody binding was detected with the Vectastain ABC Elite avidin/biotin/peroxidase kit (Vector Laboratories, Burlingame, CA, USA) for 30 min at room temperature, followed by incubation with the peroxidase substrate solution, diaminobenzidine. The tissue sections were counterstained with hematoxylin, dehydrated, mounted, and observed under light microscopy with a ×400 objective.

### Biochemical analysis

Plasma Ang II, Ang-(1–7), and aldosterone were measured as previously described [46].

### LV myocardial ACE activity assay

Solubilized LV membranes were used to determine cardiac ACE activity as previously described [47]. ACE activity was analyzed by measuring the amount of Ang II products generated after exposure of ^125^I-Ang I in the presence of all RAS inhibitors [lisinopril for ACE, SCH39370 for neprilysin, MLN-4760 for ACE2 and chymostatin for chymase, each 50 µM] plus other inhibitors [amastatin (10 µM) and bestatin (50 µM) for aminopeptidase, benzyl succinate (50 µM) for carboxypeptidase and p-chloromercuribenzoate (250 µM) for protease] and in the absence of specific enzyme inhibitors for ACE (minus lisinopril only). Enzyme activities were reported as fmoles of Ang II product formation from ^125^I-Ang I substrate per min per mg protein [47].

### Statistical analysis

All results are reported as mean ± SEM. For all endpoints, one-way ANOVA was used to determine the significance of differences among groups. Significance of interactions between groups was determined using *Tukey* post-*hoc* tests. Differences for all tests were considered significant at *p*<0.05. Analyses were performed using GraphPad Prism, version 5 (GraphPad, San Diego, CA, USA).

## Results

Significant reduction of serum estradiol levels in OVX-rats compared to sham-operated littermates confirmed the efficacy of surgical bilateral oophorectomy (Figure 1). Four weeks of subcutaneous 17β-estradiol supplementation to OVX-rats increased plasma estradiol levels to 37 pg/mL, a value that falls within the physiological range of cycling rodents [13], [48], [49]. Our previous study found substantial increases in systolic blood pressure by 8 weeks of age in OVX-rats compared to intact littermates, with a subsequent plateau at about 165 mmHg by 11 weeks [18]. The blood pressure rise and level of the hypertensive plateau in the rats with estrogen treatment from 11 to 15 weeks of age was not different from that of the OVX-vehicle treated rats. However, E2 treatment for 4 weeks attenuated the adverse effects of estrogen loss on heart weight and cardiac fibrosis, and significantly improved myocardial relaxation, or mitral annular velocity (e′) [18].

**Figure 2:**
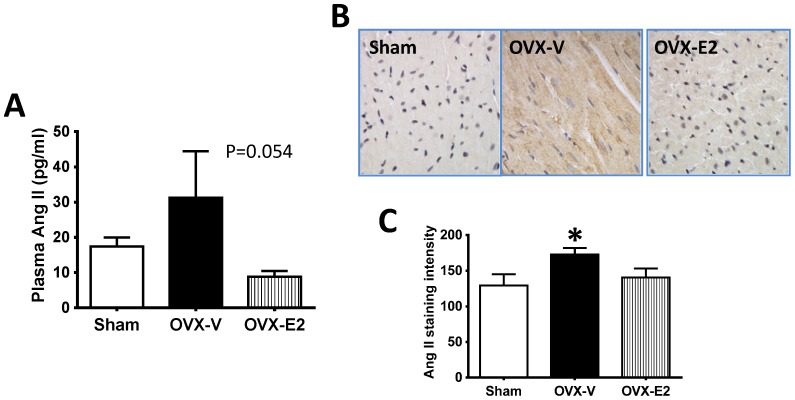
Plasma and cardiac angiotensin (Ang) II levels. (A) Plasma Ang II concentration. (B) Representative images showing Ang II staining in the left ventricles. (C) Cardiac Ang II staining intensity quantified using ImageJ. Values are mean ± SEM; * *P*<0.05 vs. sham, n = 7–13/group.

Plasma Ang II concentration tended to increase in estrogen-depleted rats compared to intact controls (*p* = 0.054), and this effect of OVX was blocked by E2 treatment (Figure 2A). Consistent with these findings, cardiac Ang II expression was increased in OVX rats, compared with intact littermates, and E2 treatment attenuated this effect (Figure 2B–C). Cardiac AT1R gene and protein expressions were not altered by either estrogen loss or E2 repletion (Figures S1).

**Figure 3:**
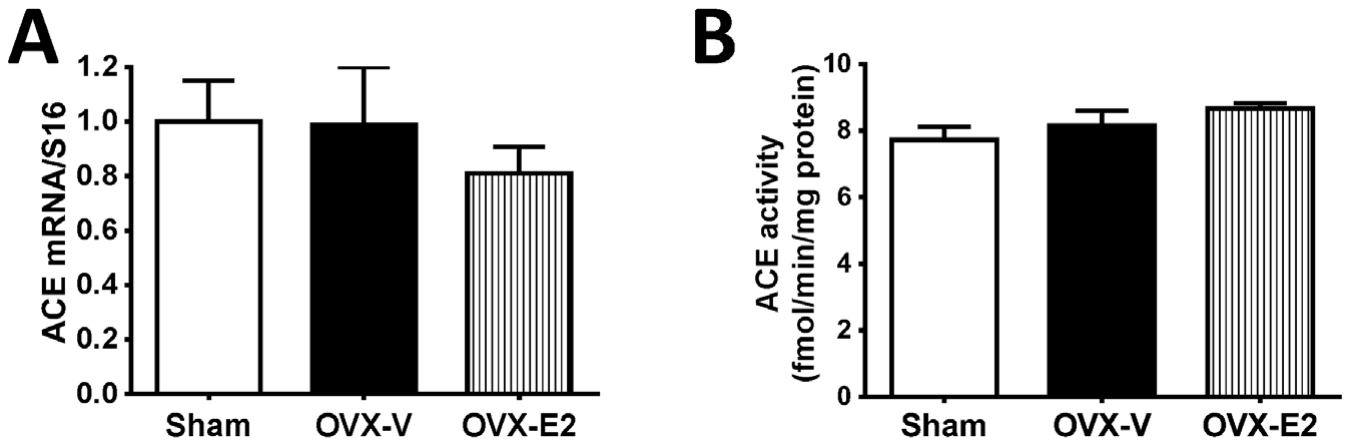
Cardiac ACE expression and activity. (A) Cardiac ACE mRNA level determined by real-time PCR, and (B) cardiac ACE activity in sham-operated and oophorectomized female mRen2.Lewis rats treated with vehicle or estradiol for 4 weeks. Values are mean ± SEM; n = 7–13/group.

The heart has all the components of the circulating RAS and, therefore, can synthesize the proteins needed to produce many of the Ang peptides. Therefore, we determined if the differential effects of estrogen status on cardiac Ang II occurred via angiotensin-converting enzyme (ACE). Interestingly, neither estrogen loss nor E2 repletion had significant effects on left ventricular ACE mRNA or ACE activity, compared to levels in hearts of intact littermates (Figure 3).

**Figure 4:**
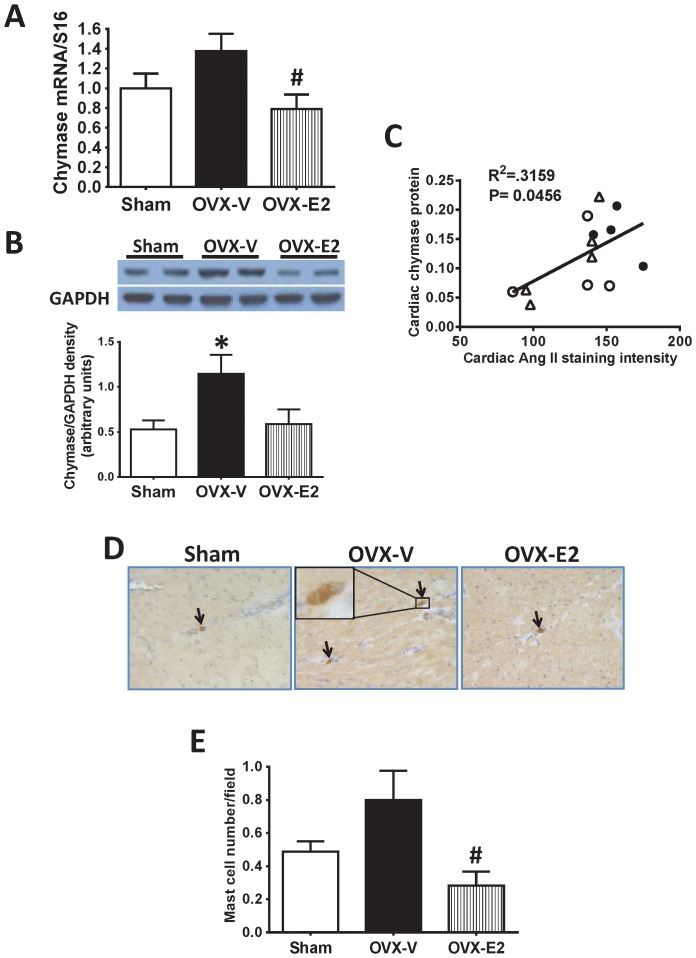
Cardiac chymase expression and mast cell number. Chymase expression and mast cell number were determined in the left ventricles of sham-operated and ovariectomized female mRen2.Lewis rats treated with vehicle or estradiol for 4 weeks. (A) Cardiac chymase mRNA level determined by real-time PCR. (B) Representative images of Western blot for chymase and the signal densities quantified using ImageJ. (C) Correlation analysis of cardiac Ang II staining intensity with cardiac chymase protein level. ○: sham, •: OVX-V, Δ: OVX-E2. (D) Representative images showing mast cell staining. (E) Quantification of cardiac mast cell number. Values are mean ± SEM; # P<0.05 vs. OVX.

Chymase, a serine protease, is another important enzyme accountable for generation of Ang II in the heart [50], [51], [52]. While chymase mRNA levels were only modestly elevated in the LV of OVX rats, chymase had a significant influence on cardiac Ang II in E2-treated OVX rats. Chymase mRNA level in the LV of OVX+E2 rats were reduced in comparison to vehicle-treated OVX littermates (Figure 4A). Cardiac chymase protein increased in OVX rats compared with sham-operated rats determined by Western blot analysis, while this increase was inhibited by E2 treatment (Figure 4B). Correlation analysis showed a strong tendency for a positive relationship between cardiac chymase protein and Ang II content (P<0.05, Figure 4C). Mast cells are the main source of chymase in the heart [44], [50], [51], [52], and immunohistochemical staining identified mast cells in the LV of the female mRen2.Lewis rats (Figure 4D). Mast cell number in OVX rats tended to be higher than in sham-operated controls, and was significantly higher in OVX rats than in E2-treated OVX littermates (Figure 4E). Thus, estrogen loss was associated with an increase in mast cell number, which was counteracted by estrogen repletion.

Upon its release from mast cells, chymase also activates MMP-9, and subsequently helps to promote tissue remodeling [53], [54]. Early surgical loss of ovarian estrogens did not overtly alter cardiac MMP-9 gene expression (Figure S2A). However, E2 repletion did significantly reduce MMP-9 mRNA levels in LV tissue, compared to levels in vehicle-treated OVX-rats (Figure S2A). Reduced MMP-9 protein expression in OVX+E2 hearts, compared to OVX hearts, confirmed this effect (Figure S2B).

**Figure 5:**
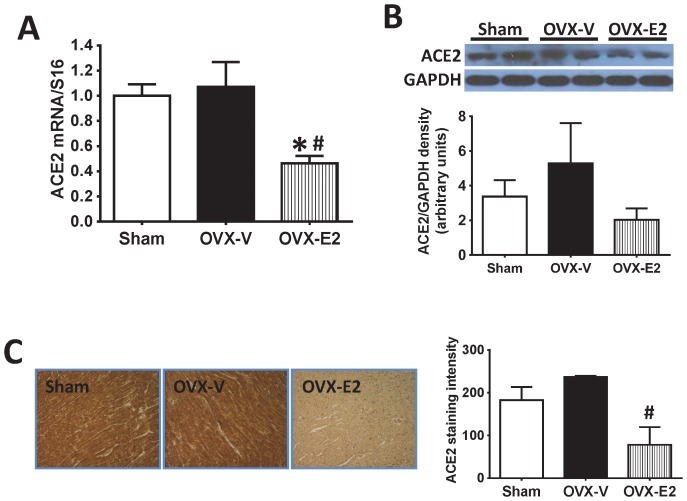
Cardiac ACE2 expression. (A) ACE2 mRNA expression determined by real-time PCR, (B) Western blot for ACE2 in left ventricles and the signal densities quantified using ImageJ, (C) Representative images showing ACE2 staining in the left ventricles, in sham-operated and oophorectomized female mRen2.Lewis rats treated with vehicle or estradiol for 4 weeks. Values are mean ± SEM; * *P*<0.05 vs. sham, # *P*<0.05 vs. OVX.

**Figure 6:**
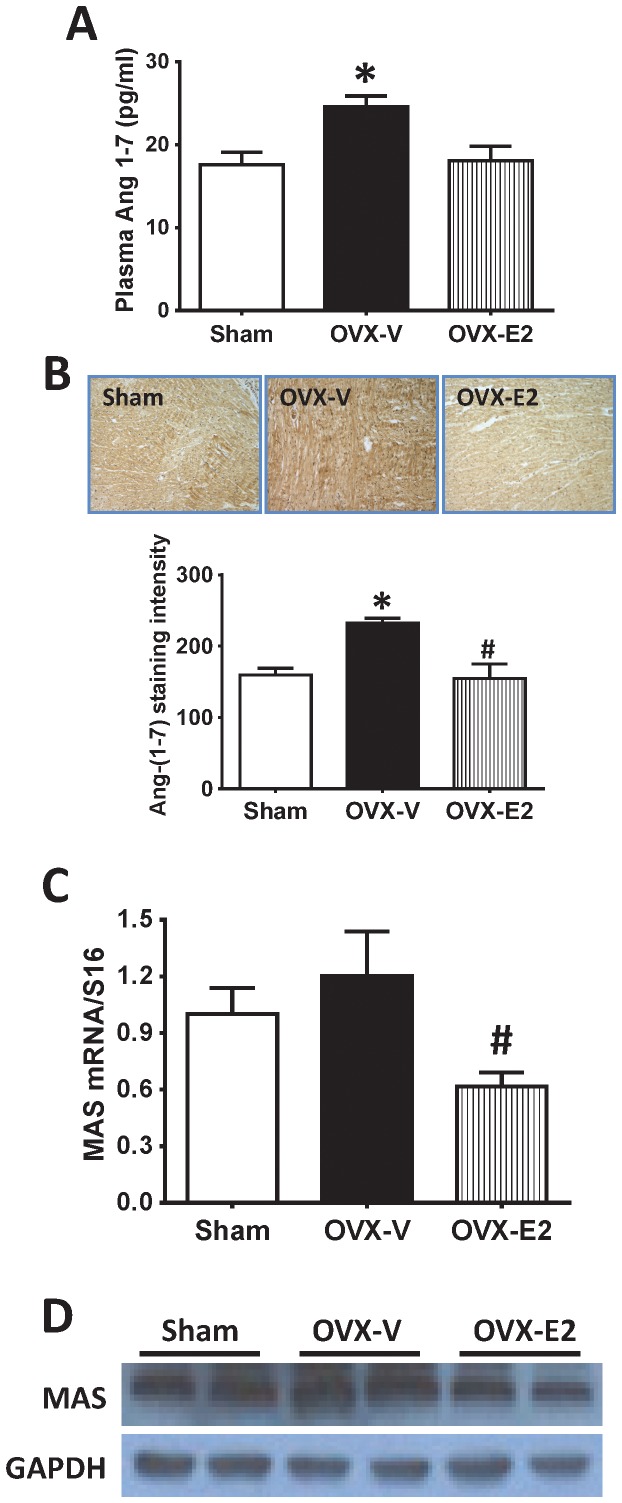
Plasma and cardiac Ang 1–7 and cardiac mas receptor. Plasma Ang 1–7, cardiac Ang 1–7 and mas receptor expression were determined in left ventricles of sham-operated and oophorectomized female mRen2.Lewis rats treated with vehicle or estradiol for 4 weeks. (A) Plasma Ang 1–7 concentration, (B) Representative images showing Ang 1–7 staining in the left ventricles and the intensities quantified using ImageJ, (C) Mas receptor mRNA expression determined by real-time PCR, (D) Western blot for mas receptor in left ventricles. Values are mean ± SEM; * *P*<0.05 vs. sham, # *P*<0.05 vs. OVX.

The ACE2/Ang-(1–7)/mas receptor arm of the cardiac RAS can act as a negative feedback regulator of the antagonistic actions of Ang II, eliciting signaling mechanisms that result in inhibition of myocyte protein synthesis and proliferation, anti-fibrotic actions, and reduced myocyte responsiveness to ischemic injury and inflammation [40], [41], [42]. Therefore, we investigated modulation of these components by estrogen. Estrogen loss did not affect ACE2 mRNA or protein levels in the LV of OVX rats (Figure 5A–C). However, E2 treatment significantly reduced cardiac ACE2 mRNA and immunohistochemistry staining, and tended to reduce cardiac ACE2 by Western blot, in comparison to OVX without estrogen replacement (Figure 5A–C). Interestingly, plasma Ang-(1–7) was significantly increased in OVX rats compared to sham-operated rats. This increase was inhibited by E2 repletion (Figure 6A). Consistent with these systemic findings, cardiac Ang-(1–7) expression was increased in the LV of OVX rats, and reduced in hearts from E2-treated OVX littermates, in comparison to sham-operated control animals (Figure 6B). Both real-time PCR and western blot analyses showed that estrogen loss by OVX did not affect cardiac expression of the Ang-(1–7) mas receptor. Conversely, E2 treatment significantly reduced cardiac mas receptor levels, compared to levels in hearts from OVX rats (Figure 6C–D).

**Figure 7:**
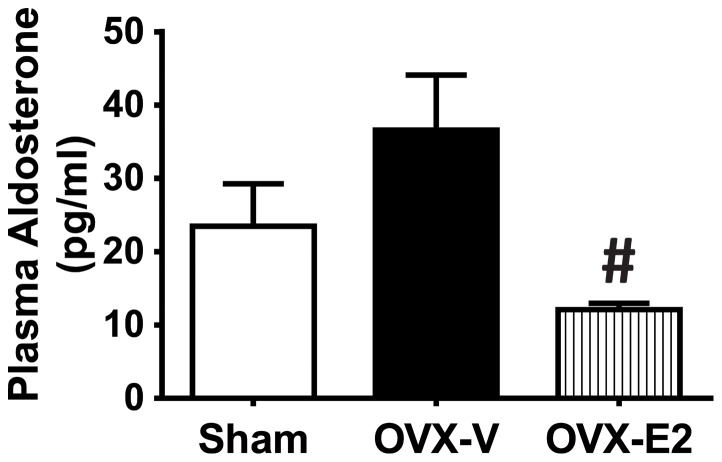
Plasma aldosterone concentration. Plasma aldosterone concentration was determined in sham-operated and oophorectomized female mRen2.Lewis rats treated with vehicle or estradiol for 4 weeks. OVX, oophorectomized. Values are means ± SEM; # *P*<0.05 vs. OVX. n = 7–13/group.

Other RAS components were also measured in this study. Renin and angiotensinogen (AO) mRNA levels in the left ventricle were determined by real-time PCR and there were no differences among sham-operated and ovariectomized female mRen2.Lewis rats treated with vehicle or estradiol for 4 weeks (Figure S3A–B). Plasma aldosterone tended to increase in estrogen-depleted rats in comparison to sham-operated rats (Figure 7). E2 treatment inhibited the OVX-related increase; aldosterone levels were significantly reduced in E2-treated OVX rats, compared to vehicle-treated OVX-rats.

## Discussion

Results of the present study provide evidence that E2 repletion might reduce the effects of OVX on local cardiac Ang II expression, by down regulating cardiac chymase and mast cell number, rather than by influencing the ACE-dependent pathway for Ang II formation. Unexpectedly, late E2 treatment, following OVX, did not appear to shift the cardiac RAS to its anti-proliferative and anti-fibrotic limb, represented by ACE2/ang-(1–7)/mas receptor.

Our previous studies showed that early OVX, at 4–5 weeks of age, resulted in exacerbated hypertension, left ventricular hypertrophy, cardiac fibrosis, and impairment of diastolic function by 15 weeks of age [13], [14], [15], [16], [17], [18]. We recently reported that late E2 treatment, initiated 11 weeks after OVX, attenuated the effect of estrogen loss on cardiac hypertrophy, remodeling, and diastolic dysfunction, independent of blood pressure [18]. Using our rodent model, which mimics the cardiovascular phenotype of postmenopausal women, the present study suggests chymase/Ang II pathway might be involved in the E2-mediated cardioprotection.

Our results showed that surgical loss of ovarian estrogens in the mRen2.Lewis rat is associated with increases in cardiac chymase and mast cell number, and that chronic E2 replacement attenuates these effects. Chymase is part of an alternative pathway for the generation of Ang II from Ang I: more than 80% of Ang II formation in human hearts occurs through the chymase pathway [50], and similarly high percentages have been observed in other species, including dog [51], and hamster [52]. While the conversion of Ang I to Ang II by chymase might be a minor component of Ang II formation in the rodent heart [50], it was recently reported that cardiac chymase also forms Ang II using the substrate of angiotensin-(1–12) in this species [55]. Interestingly, our data showed that circulating Ang I and angiotensin-(1–12) significantly increased in OVX versus intact mRen2.Lewis rats, and these increases were inhibited by chronic treatment with G1, an agonist of a new estrogen receptor GPR30 (data not shown). In the present study, cardiac expression of renin, angiotensinogen, and ACE did not change by either OVX or E2 treatment. Chappell et al. [56] also reported that systemic renin concentration did not change by OVX in mRen2.Lewis rats. Notably, there tended to be a positive correlation between cardiac chymase and local Ang II content. Taken together, these findings suggest that E2 treatment reduced the effects of OVX on cardiac Ang II likely by down regulating cardiac chymase production.

Chymase is mainly released from cardiac mast cells [44], [50], [51], [52]. Besides forming local Ang II, chymase also affects collagen metabolism by directly activating metalloproteinase-9 (MMP-9) to promote cardiac remodeling [53], [54]. Increased numbers of mast cells have been reported in explanted human hearts with dilated cardiomyopathy, and in animal models of experimentally induced hypertension, myocardial infarction, and volume overload-induced cardiac hypertrophy [44]. Despite previous studies of estrogen effects on non-cardiac mast cells [57], knowledge of the effects on cardiac mast cells and chymase is very limited. Chancey and colleagues [58] found that mast cell degranulation resulted in reduced collagen volume fraction and ventricular dilatation in hearts of normal males and ovariectomized female rats, compared to hearts of intact and estrogen-supplemented oophorectomized females. Estrogen-related cardioprotection of the volume-stressed myocardium might be the result of an altered mast cell phenotype and/or the prevention of mast cell activation [59]. The observations in the present study, that cardiac chymase and mast cell number increased in OVX mRen2.Lewis rats, and that this effect was inhibited by estrogen treatment, provide further evidence that estrogen affects the number, composition, and/or release of mast cells in the heart, ultimately affecting cardiac remodeling and function.

Animal studies showed that chronic estrogen replacement reduces ACE activity and mRNA levels in kidney and aorta extracts, with an associated reduction in plasma Ang II [60], [61]. Decreased serum ACE activity has also been observed in postmenopausal women on hormone replacement therapy [62], [63]. In the present study, E2 treatment for four weeks did not change ACE mRNA or activity in the hearts of OVX mRen2.Lewis rats. 17β-estradiol downregulated ACE mRNA in the kidney, but did not affect ACE mRNA in the lung of the same animals [64]. Thus, the effects of E2 on ACE appear to be tissue-specific. No estrogen response element was reported in the 5′ flanking region of the ACE coding sequence; however, the ACE promoter does contain a consensus AP1 site [65], [66], [67], [68]. Together, these results suggest that changes in ACE expression and activity occur in response to the local, tissue-specific environment, rather than as a direct effect of estrogen.

The AT1R mediates most of the deleterious effects of Ang II. Overexpression in cardiomyocytes or prolonged activation of AT1R causes cardiac hypertrophy and interstitial fibrosis [31], [32]. Although estrogen downregulates AT1 receptors in the kidney, pituitary, adrenal, and smooth muscle cells [24], [69], [70], [71], [72], studies of E2 modulation of cardiac AT1R have produced inconsistent results. In our mRen2.Lewis model, neither OVX nor E2 repletion caused changes in expression of cardiac AT1R genes or proteins. Similarly, Shenoy [73] found that E2 had no effect on the protein levels of cardiac AT1R in DOCA-salt hypertensive rats, and van Eickles et al. [74] observed that E2 treatment did not affect the AT1R in a mouse model of pressure overload cardiac hypertrophy. However, Ricchiuti et al. [75] did find increased levels of cardiac AT1R in E2-replaced OVX rats consuming a high-sodium diet. Thus, regulation of AT1R by estrogen might depend on the specific animal model, as well as the local tissue environment [76], or the existence of other regulatory influences such as a high salt intake.

There is increasing evidence that ACE2/Ang-(1–7) are the RAS components that oppose the actions of Ang II in the heart by acting in an antiproliferative, antiarrhythmic, anti-fibrotic, and anti-hypertrophic manner [40], [41], [42]. Administration or targeted overexpression of Ang-(1–7) in the heart prevented cardiac hypertrophy and fibrosis induced by Ang II [40], [43], which were mediated by Ang-(1–7) mas receptor [42], [77]. ACE2 is the critical enzyme that hydrolyzes Ang II into Ang-(1–7) in the heart [33], [34], [35]. ACE2 overexpression in the heart prevented Ang II-induced cardiac hypertrophy and fibrosis [36], [37], while ACE2 gene deletion in mice resulted in cardiac systolic dysfunction and LV wall thinning [38]. Chronic ACE2 inhibition led to cardiac hypertrophy and fibrosis in male Ren2.Lewis rats [39]. The regulation of estrogen on cardiac ACE2 has recently been explored in various animal models. Shenoy et al. [73] observed that a higher dose of E2 therapy caused a significant increase in cardiac ACE2 protein in DOCA-salt rats. In the present study, cardiac ACE2 levels in mRen2.Lewis rats were not affected by estrogen loss via OVX. Although the reduction of cardiac ACE2 protein expression by E2 did not reach statistical significance in the Western blot analysis, perhaps due to the small sample size (n = 5) or antibody sensitivity, E2 treatment significantly reduced cardiac ACE2 mRNA levels and the intensity of LV tissue expression by immunohistochemistry staining. Thus, as in regulation of AT1R, regulation of ACE2 by estrogen appears to be tissue- and model-dependent. It is unclear whether or not increases in systemic and local Ang-(1–7) represent a compensatory mechanism following the loss of estrogens. Ji et al. [78] reported that E2 replacement prevented the decrease in renal ACE2 that was induced by renal wrap hypertension. However, estradiol replacement downregulated renal ACE2 expression in ApoE−/− ovariectomized mice [76]. Our results showed that circulating and cardiac Ang-(1–7) increased in OVX rats without a concomitant change in *mas* receptor, while both Ang-(1–7) and its mas receptor were reduced in hearts of E2-replete OVX rats.

Aldosterone, another key component of the RAS, is mainly synthesized and released from adrenal gland [24]. Adverse effects of aldosterone on cardiac remodeling and heart failure have been reported in animal models and clinical studies [79], [80], [81]. In the present study, plasma aldosterone tended to increase in the OVX-mRen2.Lewis rats, but decreased significantly following estrogen treatment in comparison to vehicle-treated OVX rats. Estrogen might regulate aldosterone synthesis and secretion via downregulation of AT1R in the adrenal gland. Animal studies showed that estrogen decreases the number of AT1 receptors in the adrenal gland, and attenuates acute Ang II-induced aldosterone release from the adrenal zona glomerulosa [24]. Further studies are warranted to determine if E2-mediated modulation of Ang II-stimulated aldosterone secretion plays a significant role in the cardioprotective effects observed in the mRen2.Lewis model.

### Limitations of the present study

One limitation of the present study is that local RAS components were analyzed only in heart tissue of the experimentally treated mRen2.Lewis rats. Although there is increasing evidence that the local cardiac RAS plays important roles in cardiovascular disease, and that as much as 75% of cardiac Ang II is synthesized *in situ* in animal models [82], we cannot exclude the possibility that the observed increases in cardiac Ang II and Ang-(1–7) were a consequence of increased systemic levels. *In vitro* studies are needed to confirm the origin and role of cardiac Ang II and Ang-(1–7) in the postmenopausal cardiac phenotype. Second, although immunohistochemistry staining is commonly used to detect the tissue content of protein and small peptides, more accurate and sensitive biochemical methods, such as radioimmunoassay for Ang II [14], [21], [22], [61], and chymase activity assay, are needed in future studies to confirm our findings. A third limitation is that the mRen2.Lewis female is a renin-overexpressed animal model, which might not accurately emulate the RAS-related changes in women after menopause since the renin 2 gene is expressed in cardiac myocytes. The renin 2 gene will not be expressed in the human heart and a question remains as to whether renin is expressed in human cardiomyocytes. Moreover, although E2 treatment decreased cardiac expression of MMP-9, ACE2, and mas receptor in OVX rats, there were no differences in the expression of those genes or proteins between OVX and sham-operated animals. The heightened baseline expression of the RAS in transgenic mRen2.Lewis rats might explain the lack of significant change in RAS components following OVX alone.

The present study, using the estrogen-sensitive mRen2.Lewis rat, provides intriguing evidence that exogenous E2 might have a role in modulating the local cardiac RAS viadownregulating cardiac chymase expression. Although E2 treatment in this study did not overtly affect systemic blood pressure, it is unclear whether subtle changes in endothelial structure and function by E2 replacement had a direct impact on local RAS production or dynamics. Also, additional studies are underway to determine the exact roles of cardiac chymase and RAS in estrogen-related changes in myocyte size, vascular content, inflammatory cell infiltration, cardiac fibrosis and heart function.

## Supporting Information

Figure S1 **Cardiac AT1R expression.** (A) AT1R mRNA level determined by real-time PCR, and (B) Representative images showing Western blot for AT1R in sham-operated and ovariectomized female mRen2.Lewis rats treated with vehicle or estradiol for 4 weeks. Values are mean ± SEM; n = 7–13/group.
(TIF)

Figure S2 **Cardiac MMP-9 expression.** Cardiac MMP-9 expression was determined in the sham-operated and ovariectomized female mRen2.Lewis rats treated with vehicle or estradiol for 4 weeks. (A) MMP-9 mRNA level determined by real-time PCR. (B) Representative images showing Western blot for MMP-9. Values are mean ± SEM; # *P*<0.05 vs. OVX.
(TIF)

Figure S3 **Cardiac renin and AO expression.** Renin (A) and AO (B) mRNA levels in left ventricles determined by real-time PCR in sham-operated and ovariectomized female mRen2.Lewis rats treated with vehicle or estradiol for 4 weeks. Values are mean ± SEM; n = 7–13/group.
(TIF)
