# Supplementary figures and images for: Characterization of the Cardiac Renin Angiotensin System in Oophorectomized and Estrogen-Replete mRen2.Lewis Rats
Source: PLoS One. 2013 Oct 25;8(10):e76992. doi: 10.1371/journal.pone.0076992 (PMC3808369; doi:10.1371/journal.pone.0076992)

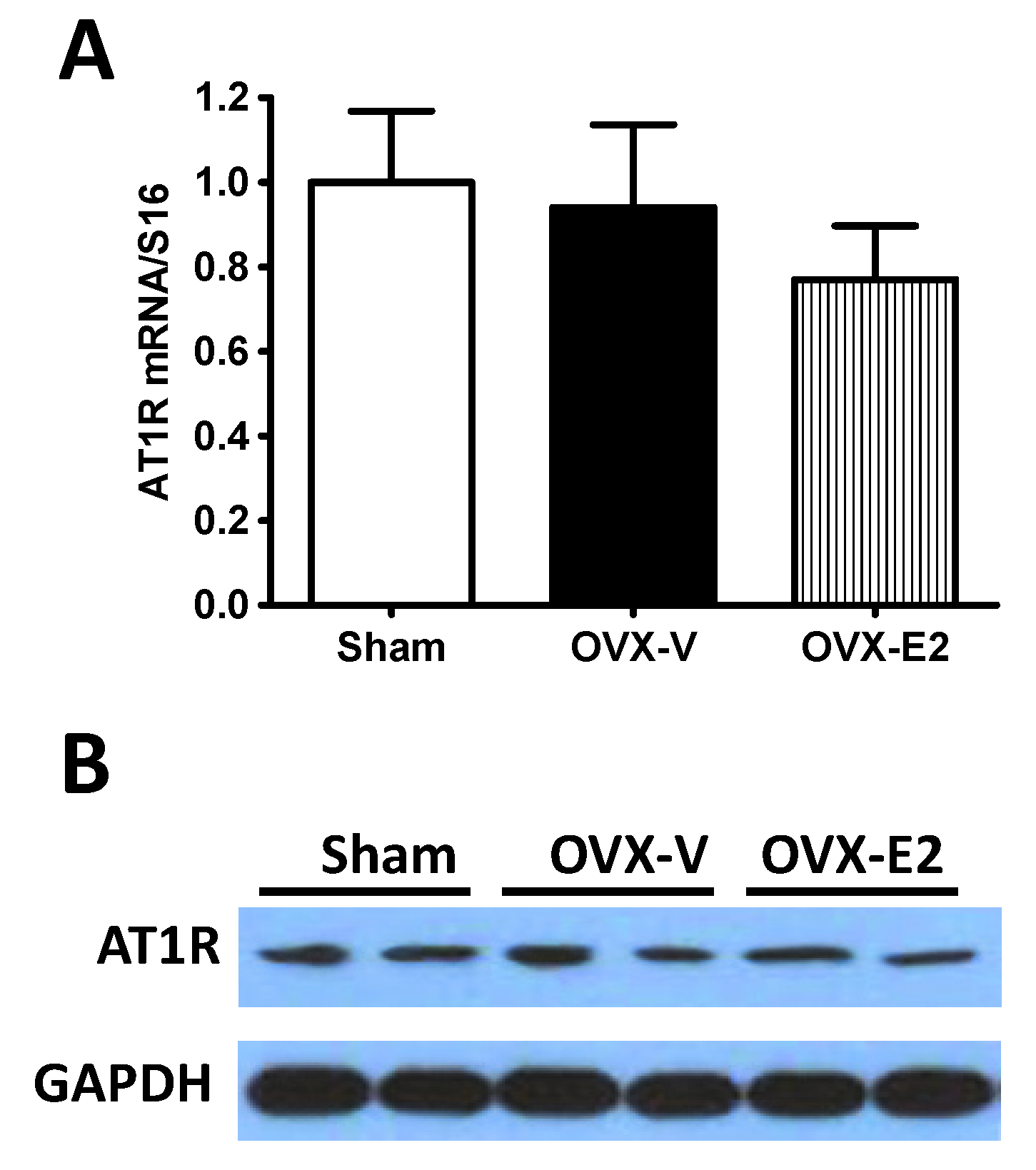

Supplement: Figure S1 — Cardiac AT1R expression. (A) AT1R mRNA level determined by real-time PCR, and (B) Representative images showing Western blot for AT1R in sham-operated and ovariectomized female mRen2.Lewis rats treated with vehicle or estradiol for 4 weeks. Values are mean ± SEM; n = 7–13/group. (TIF) [file pone.0076992.s001.tif]

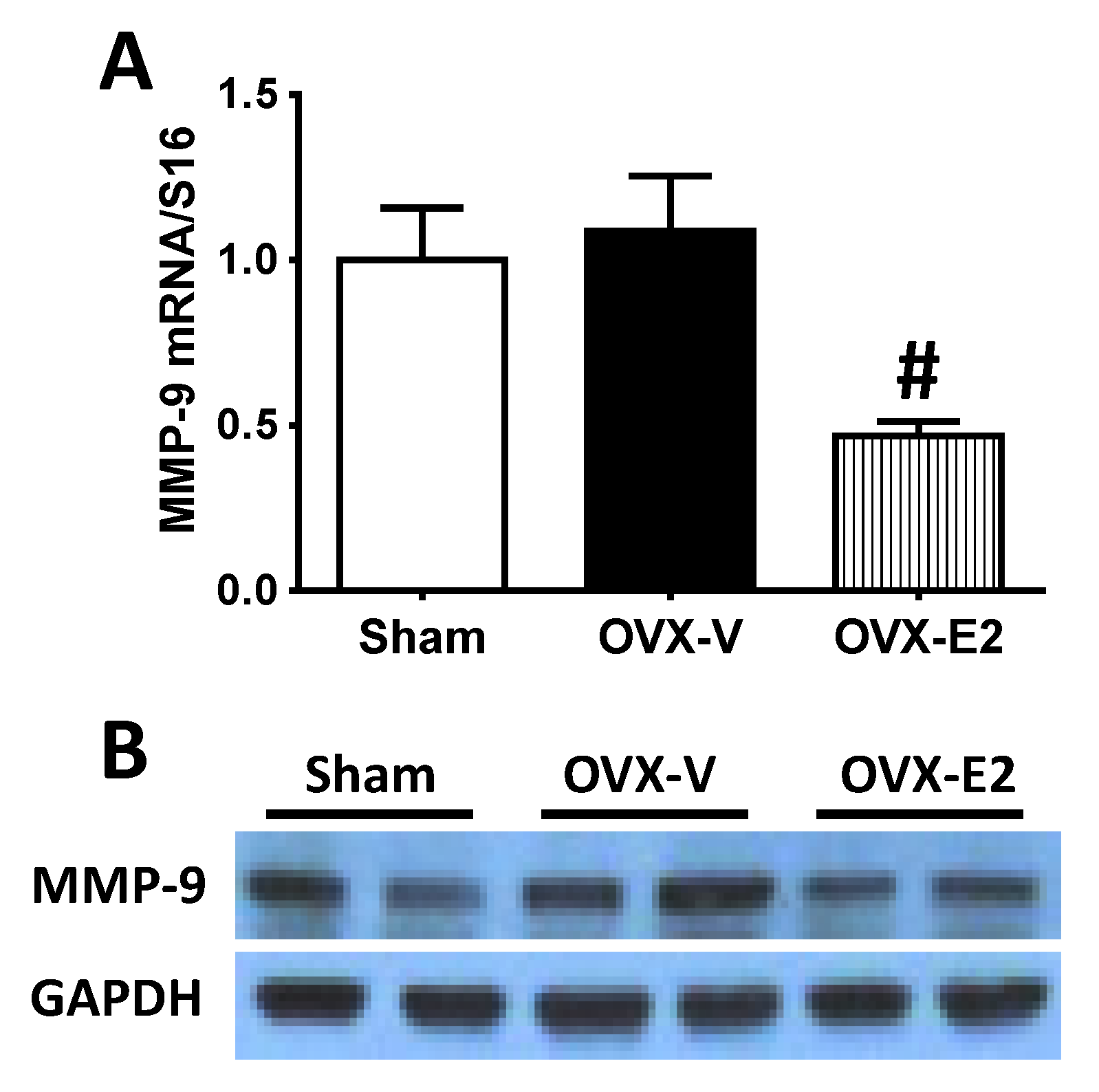

Supplement: Figure S2 — Cardiac MMP-9 expression. Cardiac MMP-9 expression was determined in the sham-operated and ovariectomized female mRen2.Lewis rats treated with vehicle or estradiol for 4 weeks. (A) MMP-9 mRNA level determined by real-time PCR. (B) Representative images showing Western blot for MMP-9. Values are mean ± SEM; # P<0.05 vs. OVX. (TIF) [file pone.0076992.s002.tif]

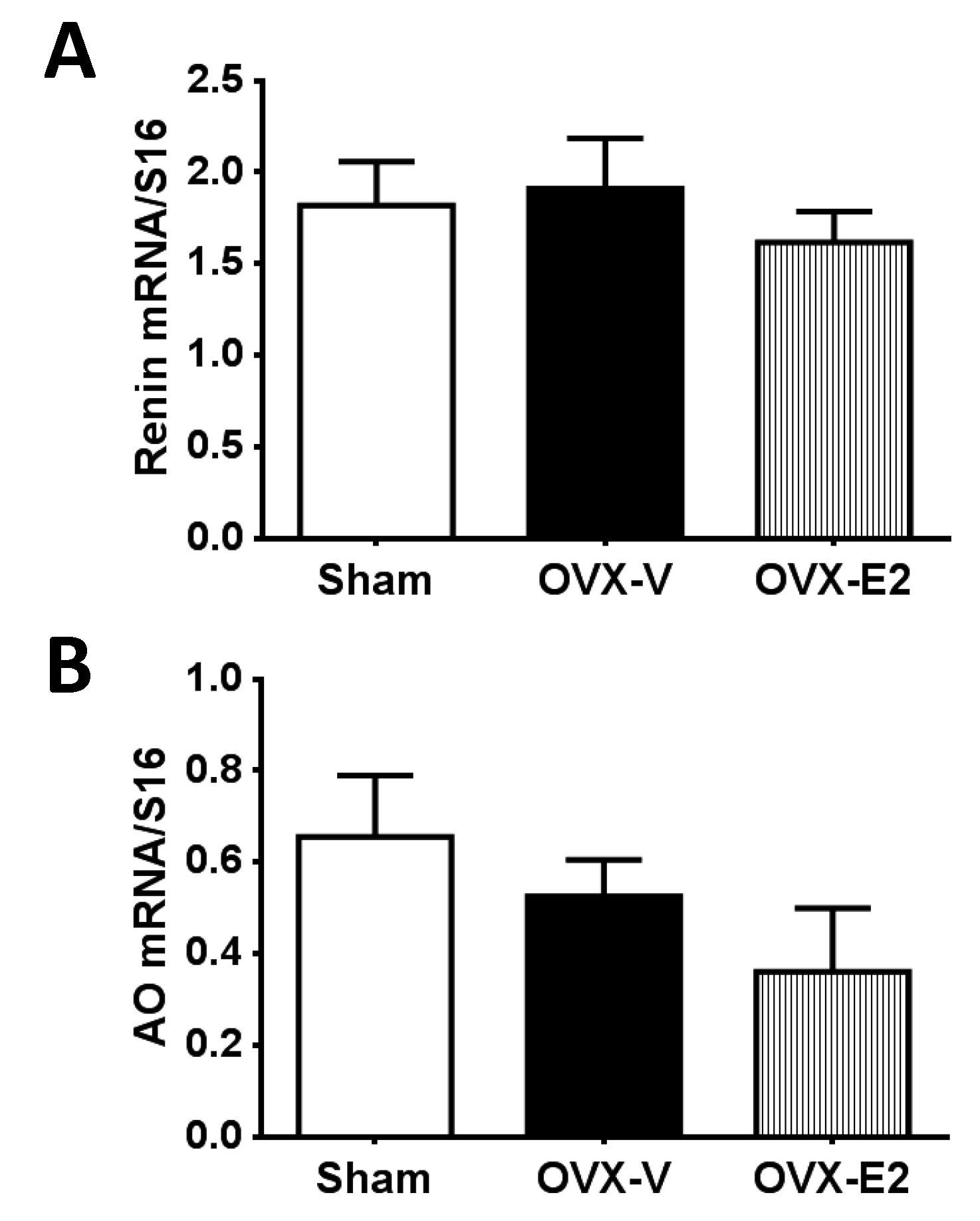

Supplement: Figure S3 — Cardiac renin and AO expression. Renin (A) and AO (B) mRNA levels in left ventricles determined by real-time PCR in sham-operated and ovariectomized female mRen2.Lewis rats treated with vehicle or estradiol for 4 weeks. Values are mean ± SEM; n = 7–13/group. (TIF) [file pone.0076992.s003.tif]
